# Effect of incorporation of broccoli residues into soil on occurrence of verticillium wilt of spring-sowing-cotton and on rhizosphere microbial communities structure and function

**DOI:** 10.3389/fbioe.2023.1115656

**Published:** 2023-01-24

**Authors:** Weisong Zhao, Peipei Wang, Lihong Dong, Shezeng Li, Xiuyun Lu, Xiaoyun Zhang, Zhenhe Su, Qinggang Guo, Ping Ma

**Affiliations:** Integrated Pest Management Innovation Center of Hebei Province, Key Laboratory of IPM on Crops in Northern Region of North China, Institute of Plant Protection, Hebei Academy of Agriculture and Forestry Sciences, Ministry of Agriculture and Rural Affairs, Baoding, China

**Keywords:** spring-sowing-cotton, verticillium wilt, broccoli residues, microbial community structure, function, rhizosphere

## Abstract

Cotton verticillium wilt (CVW) represented a typical plant soil-borne disease and resulted in widespread economic losses in cotton production. However, the effect of broccoli residues (BR) on verticillium wilt of spring-sowing-cotton was not clear. We investigated the effects of BR on CVW, microbial communities structure and function in rhizosphere of two cotton cultivars with different CVW resistance using amplicon sequencing methods. Results showed that control effects of BR on CVW of susceptible cultivar (cv. EJ-1) and resistant cultivar (cv. J863) were 58.49% and 85.96%, and the populations of *V. dahliae* decreased by 14.31% and 34.19%, respectively. The bacterial diversity indices significantly increased in BR treatment, while fungal diversity indices significantly decreased. In terms of microbial community composition, the abilities to recruit bacteria and fungi were enhanced in BR treatment, including *RB41*, *Gemmatimonas*, *Pontibacter*, *Streptomyces*, *Blastococcus*, *Massilia*, *Bacillus*, and *Gibberella*, *Plectosphaerella*, *Neocosmospora*, *Aspergillus* and *Preussia*. However, the relative abundances of *Sphingomonas*, *Nocardioides*, *Haliangium*, *Lysobacter*, *Penicillium*, *Mortierella* and *Chaetomidium* were opposite tendency between cultivars in BR treatment. According to PICRUSt analysis, functional profiles prediction showed that significant shifts in metabolic functions impacting KEGG pathways of BR treatment were related to metabolism and biosynthesis. FUNGuild analysis indicated that BR treatment altered the relative abundances of fungal trophic modes. The results of this study demonstrated that BR treatment decreased the populations of *V. dahliae* in soil, increased bacterial diversity, decreased fungal diversity, changed the microbial community structure and function, and increased the abundances of beneficial microorganisms.

## Introduction

Cotton (*Gossypium hirsutum L*.) is the most important source of natural textile fibers worldwide and a significant oilseed crop (Y.L. Zhang et al., 2016). In China, cotton planting patterns are divided into spring-sowing-cotton and summer-sowing-cotton. Cotton growing areas mainly include Northwest inland cotton region, Yellow River basin cotton region and Yangtze River basin cotton region. In Northwest inland cotton region and Yellow River basin cotton region, spring-sowing-cotton is the main planting pattern, while summer-sowing-cotton is the main planting pattern in Yangtze River basin cotton region. As a single season crop, spring-sowing-cotton is generally late maturing cultivars with a long growth period. Summer-sowing-cotton is early maturing cultivars and short growth period. Summer-sowing-cotton was sown in late May to early June. The main planting patterns of summer-sowing-cotton are wheat-cotton double cultivation, oilseed rape-cotton double cultivation, cabbage-cotton double cultivation and so on. Broccoli is planted and an important economic crop in Hebei province. There are generally two planting patterns for broccoli in this area, one is planted in early spring (March), and the other in summer and autumn (early August). We have previously evaluated the impact of BR on verticillium wilt of summer-sowing-cotton (W.S. Zhao et al., 2019).

Cotton verticillium wilt (CVW), caused by *Verticillium dahliae*, is a typical soil-borne disease and results in extensive economic losses. In China, losses of approximately 250–310 million US dollars have been reported for cotton annually due to *V. dahliae* (C.H. Li et al., 2015; L; Rehman et al., 2018). CVW is particularly difficult to control due to the long-living dormant microsclerotia produced by the pathogen, which could remain viable in the soil for more than 2 decades (E.F. Fradinand Thomma, 2006; S; Alstrom 2001), as well as the inability of fungicides to contact the hyphae of *V. dahliae* after they spread inside the xylem (S.J. Klosterman et al., 2009). It is imperative to develop novel control strategies to control this devastating disease. Previous studies showed that soil-borne disease management has relied principally upon fumigation (Z.K. Atallah et al., 2012; D.A; Johnson and Dung, 2010; R.J; Taylor et al., 2005). However, the application of chemical fumigants to the soil may be environmentally unfriendly (A.K. Uppal et al., 2008). Therefore, there is growing interest in the search for alternatives to fumigants for disease control. Many reports have demonstrated that the use of organic soil amendments may be a potential strategy for the control of insect pests, pathogens, nematodes, and weeds (J.C. Díaz-Pérez et al., 2012; J.A; López-Pérez et al., 2005; J; Poveda et al., 2021), since they could increase organic matter and nutrients and change the structure of the microbial community in the soil (Z.Z. Shen et al., 2013; S; Akao 2017; D.L; Chen et al., 2022). The changes in soil microbial community structure caused by organic soil amendments provide useful information on soil health and quality (P.H.B. Poulsen et al., 2013; N; Rodríguez-Berbel et al., 2022). In particular, the responses of soil bacterial communities to organic soil amendments are particularly important and are believed to be one of the main drivers of disease suppression (P. Garbeva et al., 2004; A.O; Akanmu et al., 2021). Broccoli residues (BR) were reported to successfully reduce the incidence of verticillium wilt in eggplant, potato, strawberry, sunflower and summer-sowing-cotton by reducing microsclerotia or DNA copy numbers of *V. dahliae* in the soil (P. Inderbitzin et al., 2018; W.S; Zhao et al., 2019; 2021). Mechanism for control was partially attributed to breakdown of glucosinolates from BR in soil to produce volatile compounds (isothiocyanates) that had fungicidal properties (G.D. Bending and Lincoln, 1999; K.V; Subbarao et al., 1999). However, more in-depth research should be performed to further explore this potential mechanism, especially from the perspective of rhizosphere microbiomics. Microbiome-based research has opened a new frontier that will greatly expand our knowledge of the relationships between plant disease incidence and microbiota, and offer new opportunities for developing novel approaches for biocontrol. To our knowledge, this detailed comparison of the soil microbial community associated with different cultivars resistant to CVW was the first to be performed in BR treatment.

Hebei is the second largest cotton growing province in China, the planting area is approximately 150 thousands hectares, with spring-sowing-cotton as the main planting pattern. In our previous study, it was showed that BR could reduce the occurrence of summer-sowing-cotton verticillium wilt and improve soil bacterial community structure under the broccoli-cotton double cropping cultivation mode (W.S. Zhao et al., 2019). However, the effects of BR on the occurrence of verticillium wilt of spring-sowing-cotton and soil bacterial and fungal communities are still unclear.

Recent evidences suggest that variability in plant genotypes or cultivars could have a significant impact on rhizosphere microbiomes, particularly bacteria (I.A. Stringlis et al., 2018; J.Y; Zhang et al., 2019). However, information regarding the variation in soil microbial communities in rhizosphere that are influenced by cotton cultivars that vary in resistance to CVW following the application of BR is still lacking. It is unclear whether and, if so, how cultivar resistance against *V. dahliae* is related to rhizosphere microorganisms. The overall objectives of this study were therefore i) to determine the effect of BR on the incidence of verticillium wilt of spring-sowing-cotton with different CVW resistance levels, ii) to study the differences in soil microbial diversity indices, and microbial communities structures in BR treatment, iii) to analyze the effect of BR on soil microbial function.

## Materials and methods

### Field experiment site

The experimental sites were located in Quzhou County (115°01′E, 36°47′N), Hebei Province. Field had a same long history of cotton cultivation and incidence of CVW. The field with flat terrain, relatively uniform fertility and continuous cotton planting for more than 10 years. Soil nutrient characteristics were described as follows: soil organic matter 0.66%, nitrate nitrogen 21.3 mg/kg, ammonium nitrogen 12.5 mg/kg, available potassium 0.25 mg/kg, available phosphorus 0.68 mg/kg, and pH 8.35.

### Experimental setup and design

The experiment was carried out from 2018 to 2019. The field was divided into two plots. The first plot was named BR, which broccoli (cultivar Yanxiu) was planted in August 2018, after edible portion was harvested, then the incorporation of BR into the soil was carried out according to the method of previous study (W.S. Zhao et al., 2020), then following planted by cotton (2019). The second plot was named CK, cotton was planted for two consecutive years (2018 and 2019). Cotton cultivar Ejing 1 (EJ-1, susceptible for CVW) and cultivar Ji 863 (J863, resistant for CVW) with different CVW resistance levels were planted on 25 April. The experimental design included four treatments: 1) susceptible cultivar EJ-1 planted without BR (EJ-1-CK); 2) susceptible cultivar EJ-1 planted with BR (EJ-1-BR); 3) resistant cultivar J863 planted with BR (J863-BR); 4) resistant cultivar J863 planted without BR (J863-CK). Experiment consisting of thirty-six 40 m long rows in field, spaced 75 cm in per row, per plot with three rows represented a replicate, each replicate area was 60 square meters. The experiment was three replicates.

### Soil sample collection, DNA extraction and qPCR analysis for *V. dahliae*

Soil samples from different treatments were collected at the flowering and boll-forming stages of cotton. Within each sampling plot, three plants were randomly selected and carefully removed from the soil using a spade. The root systems of the three plants from each plot were first vigorously shaken to remove loosely adhering soil particles, and then the remaining root systems were combined as a rhizosphere sample. Soil samples were immediately preserved at 4°C for less than 48 h. To remove plant material, samples were sieved through a 2.0 mm sieve and stored at −80°C for subsequent DNA extraction. DNA from samples were extracted following the instruction manual for the FastDNA™ SPIN Kit for Soil (MP Biomedicals, Solon, OH, United States) in accordance with the protocol of the manufacturer. The concentration and quality of DNA were determined using a spectrophotometer (NanoDrop 2000; Thermo Fisher Scientific Inc., Waltham, MA, United States). The extracted DNA was stored at −20°C prior for further analyses. The DNA copy numbers of *V. dahliae* in different soil samples were determined through qPCR according to the method described in our previous study (W.S. Zhao et al., 2019).

### PCR amplification, Illumina MiSeq sequencing and data processing

PCR amplification of bacterial 16 S rRNA targeting the V3/V4 region was conducted by using primers 338 F (5′-ACT​CCT​ACG​GGA​GGC​AGC​A-3′) and 806 R (5′-GGACTACHVGGGTWTCTAAT-3′), and fungal ITS-1 region with the primers ITS1F (5′-CTT​GGT​CAT​TTA​GAG​GAA​GTA​A-3′) and ITS2R (5′-GCT​GCG​TTC​TTC​ATC​GAT​GC-3′) (W.S. Zhao et al., 2021). PCR protocols were used to amplify the 16 S rRNA gene and ITS gene (W.S. Zhao et al., 2021; R; Lu et al., 2018). Finally, equal amounts of PCR product from each sample were placed in individual tubes and analyzed with the Illumina MiSeq platform. Illumina MiSeq sequencing was performed at Majorbio Biopharm Technology Co., Ltd. (Shanghai, China). The raw sequences data were deposited at the National Center for Biotechnology Information (NCBI) under accession numbers PRJNA734729 and PRJNA894483. Processing of the raw sequences were performed using the QIIME 1.9 software. Paired-end reads were assigned to samples based on their unique barcode and were merged using FLASH 1.2 software. Reads (average quality score <20), improper primers and ambiguous bases were discarded before clustering (Y.X. Feng et al., 2019). The effective sequences were clustered into operational taxonomic units (OTUs) at 97% similarity using UPARSE 7.0 software. Soil bacterial and fungal diversity indices were calculated based on resampled OTU abundance matrices in MOTHUR 1.30 software.

### Assessment the effects of BR on cotton growth promotion and CVW incidence

The effects of BR on cotton growth-related traits were investigated. CVW severities of all individual plants were recorded on a scale of 0–4. The disease index of CVW for each plot was calculated based on a five-level categorization of CVW according to the percentage of plant leaves with symptoms such as chlorosis, necrosis or defoliation (W.S. Zhao et al., 2019). The detailed statements were as follows: 0 = healthy plants or no symptoms, 1 = diseased plants with leaf symptoms below 25%, 2 = 26%–50% diseased plants with leaf symptoms and leaf margin rolled up and showing symptoms of wilt, 3 = 51%–75% diseased plants with leaf symptoms and leaf margin rolled up with wilting symptoms, 4 = more than 76% diseased plants or dead with leaf symptoms. The disease incidence, disease index and control effect were calculated using the following formula:

$$Disease\;index=\left[100\times \sum \left(No.of\;diseased\;plants\times responding\;disease\;rating\right)\right]/\left(total\;plant\;numbers\times 4\right)$$

$$Control\;effect\left(\%\right)=\left[\left(disease\;index\;of\;CK\;treatment-disease\;index\;of\;BR\;treatment\right)/disease\;index\;of\;CK\;treatment\right]\times 100$$

### Statistical analysis

Statistically significant differences (*p* < 0.05) in disease index, DNA copy numbers of *V. dahliae*, and changes in soil bacterial and fungal community composition between CK and BR treatments were evaluated with Student’s t-test or one-way analysis of variance (ANOVA) using SPSS 17.0. Principal component analysis (PCA) was performed to explore the differences in soil bacterial and fungal community structures. Analysis of similarities (ANOSIM) were performed to identify the significant differences in bacterial and fungal community structure and function among treatments. Phylogenetic Investigation of Communities by Reconstruction of Unobserved States (PICRUSt) software package was used to predict the functional composition of bacterial communities in different samples from amplicon sequencing results. The functional genes were identified from Kyoto Encyclopedia of Genes and Genomes (KEGG) database. FUNGuild database was used to analyze, classify, and interpret fungal communities according to fungal functions. The FUNGuild software annotates taxonomic data within the OTU table with corresponding data on its online database, the annotations include the guild, trophic mode and growth morphology; only confidence scores of “Probable” and “Highly Probable” were used. Graphs were generated with Origin 8.6 software.

## Results

### Effect of BR on CVW of different cultivars and on population of *V. dahliae* in rhizosphere soil

**FIGURE 1:**
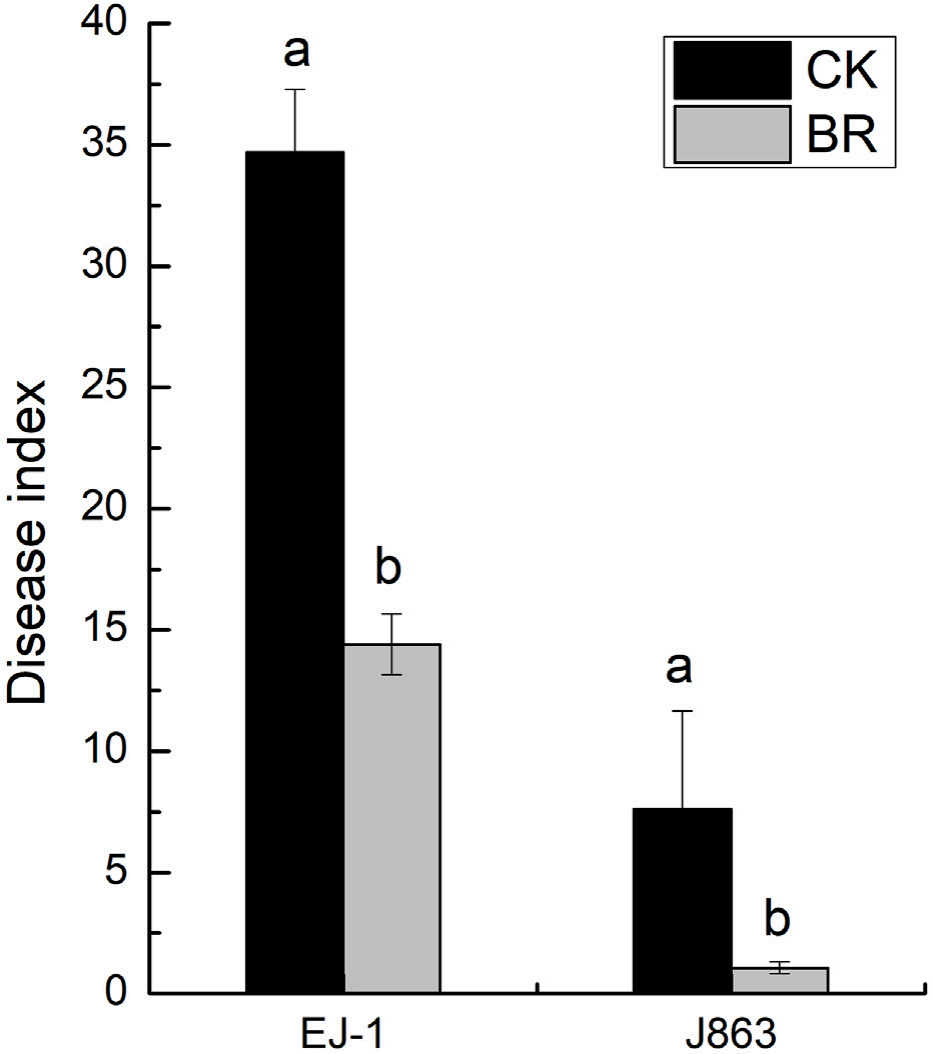
Comparison of the incidence of verticillium wilt of different cotton cultivars. CK represents treatment with blank control, BR represents treatment with broccoli residues. EJ-1 represents susceptible cultivar for CVW, J863 represents resistant cultivar for CVW. Values are the means of three replicates. Means with the same letters for the same cultivar are not significantly different according to Student’s t-test at *p* < 0.05.

**FIGURE 2:**
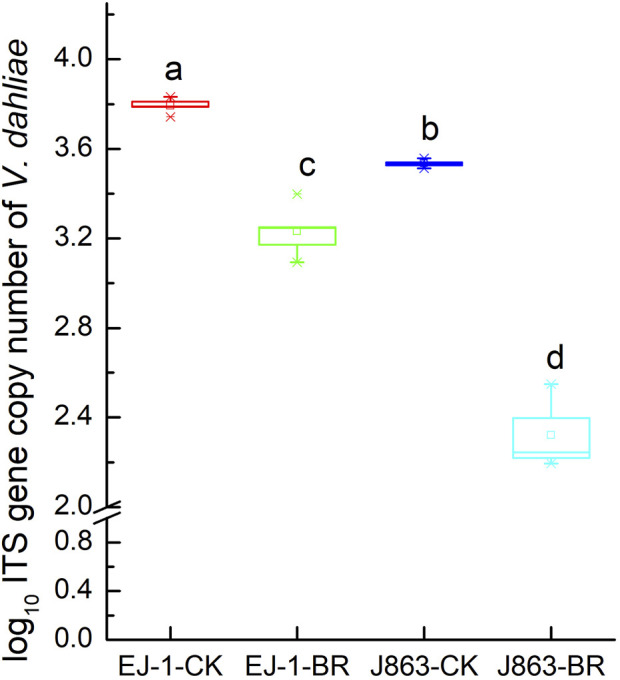
Quantification of *V. dahliae* in soil by qPCR. CK represents treatment with blank control, BR represents treatment with broccoli residues. EJ-1 represents susceptible cultivar for CVW, J863 represents resistant cultivar for CVW. Bars with different letters are significantly different by Duncan’s multiple range test at *p* < 0.05.

BR treatment had a significant impact on the disease index of CVW (*p* < 0.05). For susceptible cultivar (EJ-1), the disease indices were 34.69 and 14.40 under CK and BR treatments, respectively. For resistant cultivar (J863), the disease indices were 7.62 and 1.07 under CK and BR treatments, respectively. The disease indices of susceptible cultivar (EJ-1) and resistant cultivar (J863) were decreased by 58.49% and 85.96%, respectively (Figure 1). Compared with CK, the DNA copy numbers of *V. dahliae* in the rhizosphere from susceptible cultivar and resistant cultivars treated with BR were significantly reduced by 14.31% (*p* = 0.015) and 34.19% (*p* = 0.007), respectively (Figure 2).

### Effect of BR on cotton growth promotion

Cotton growth traits were recorded among different treatments, respectively (Table 1). For cultivar EJ-1, compared with CK treatment, all growth traits (such as plant height, ground diameter, fresh weight, dry weight, and number of branches) were significantly increased. For cultivar J863, except plant height characteristic, there were no significant differences on ground diameter, fresh weight, dry weight, and number of branches of cotton. In addition, yields of the susceptible and resistant cultivar under BR treatment were 3532.55 kgha^−1^ and 5791.98 kgha^−1^, and the yield increase rates were 8.4% and 3.7%, respectively (Table 1).

**TABLE 1:** Effect of BR on cotton growth promotion.

Cultivar	Treatment	Plant height (cm)	Ground diameter (cm)	Plant fresh weight (kg)	Dry weight (kg)	Number of branches	Yield (kg/ha)
Susceptible EJ-1	CK	107.20 (3.67) b	1.83 (0.11) b	0.94 (0.36) b	0.23 (0.04) b	12.75 (1.48) b	3259.10 (339.35) a
	BR	157.23 (1.12) a	2.37 (0.09) a	1.66 (0.35) a	0.35 (0.03) a	21.25 (5.54) a	3532.55 (190.35) a
Resistant J863	CK	122.05 (8.09) b	2.17 (0.03) a	1.09 (0.23) a	0.26 (0.05) a	13.75 (0.83) a	5587.45 (785.54) a
	BR	148.17 (3.76) a	2.17 (0.05) a	1.23 (0.21) a	0.29 (0.05) a	14.50 (0.50) a	5791.98 (796.61) a

Values are the means of three replicates. Means with the same letters are not significantly different according to Student’s t-test at *p* < 0.05. CK, represents treatment with blank control; BR, represents treatment with broccoli residues. Susceptible represents cultivar EJ-1, Resistant represents cultivar J863.

### Alpha diversity of soil bacterial and fungal communities

**FIGURE 3:**
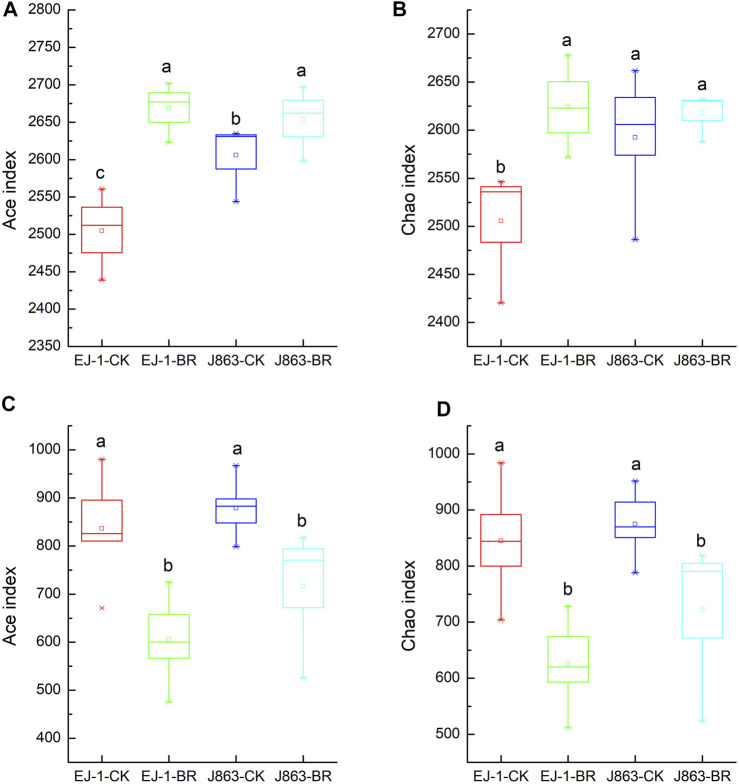
Effect of the BR treatment on the alpha diversity indices of soil bacterial and fungal community. **(A, B)** bacterial, **(C, D)** fungal. CK represents treatment with blank control, BR represents treatment with broccoli residues. EJ-1 represents susceptible cultivar for CVW, J863 represents resistant cultivar for CVW. Bars with different letters are significantly different by Duncan’s multiple range test at *p* < 0.05.

Alpha diversity of soil microbial communities were expressed by ACE and Chao indices in our study (Figure 3). For bacterial, ACE and Chao indices in different cultivars under BR treatment were higher than that in CK treatment (Figures 3A, B). However, for fungi, ACE and Chao indices in different cultivars under BR treatment were lower than that in CK treatment (Figures 3C, D).

### Beta diversity of soil bacterial and fungal community structures

**FIGURE 4:**
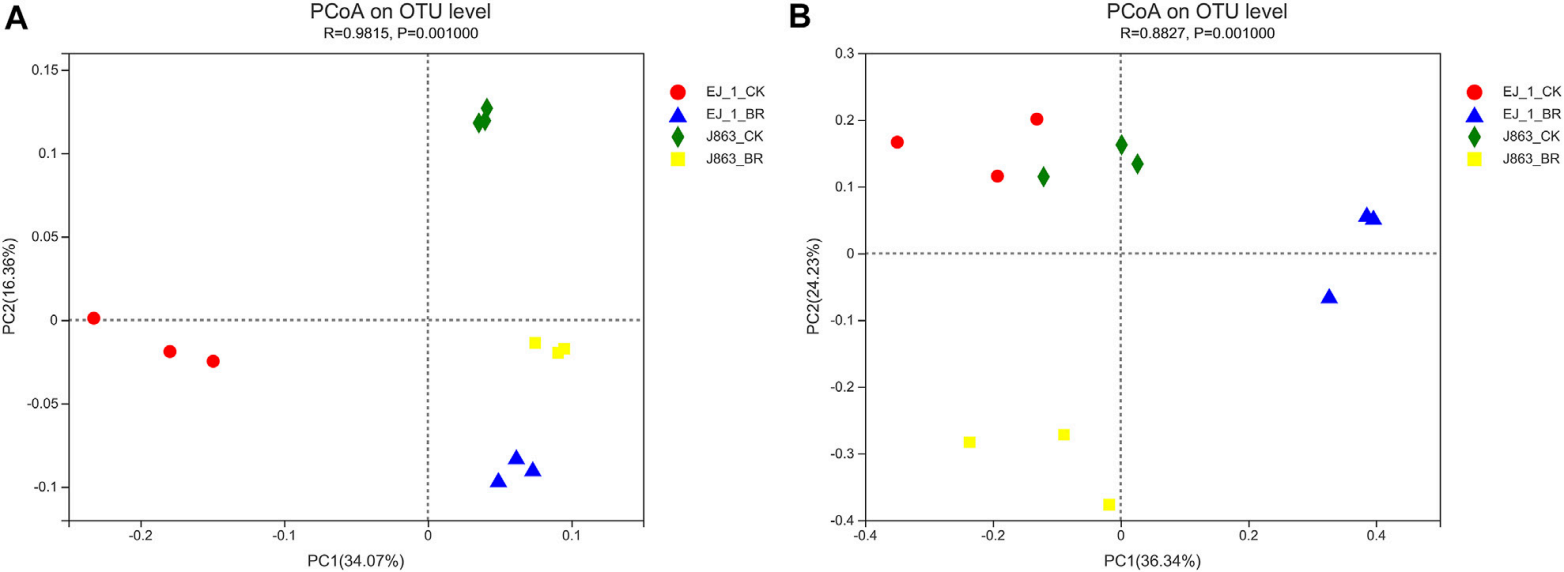
Principal component analysis (PCA) of microbial communities at the OTU level under different treatments. **(A)** bacterial, **(B)** fungal. CK represents treatment with blank control, BR represents treatment with broccoli residues. EJ-1 represents susceptible cultivar for CVW, J863 represents resistant cultivar for CVW.

Principal component analysis based on the OTU level was used to study the effects of BR on soil bacterial and fungal community structures associated with different cotton cultivars (Figure 4). The results showed that the bacterial community structures associated with the different cultivars were located in the same quadrant in BR treatment, while that of the blank control of the different cultivars were located in the different quadrants. It was indicated that bacterial community structures changed and converged together in BR treatment (Figure 4A). However, the fungal community structures between different cultivars were located in different quadrants in BR treatment, while that of the blank control of different cultivars were located in the same quadrant, which also indicated that fungal community structures changed in BR treatment (Figure 4B). In addition, the first principal component (PC1) and the second principal component (PC2) at the OTU level in rhizosphere soil were found to explain 34.07% and 16.36% of all variables in bacterial community structure, and 36.34% and 24.23% of all variables in fungal community structure, respectively. The cumulative contribution rates of variance of the two principal components reached 50.43% and 60.57%, respectively. In addition, ANOSIM indicated that BR treatment contributed significantly to the separation of CK treatment (*R* = 0.9815, *p* = 0.001, bacterial) and (*R* = 0.8827, *p* = 0.001, fungal).

### Comparison of bacterial community composition

**FIGURE 5:**
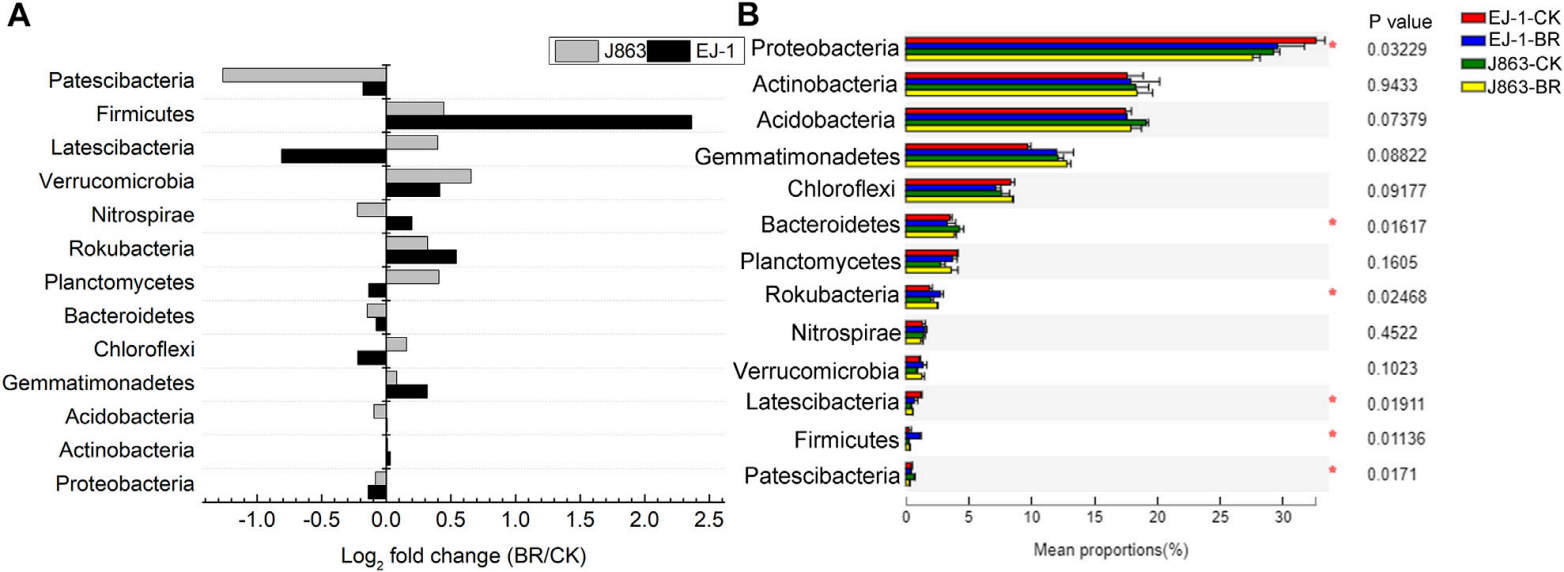
Relative abundance of the dominant bacterial taxa at the phylum level under different treatments. **(A)** Logarithm of fold changes of BR/CK, **(B)**, Analysis of significant differences in mean proportion of dominant bacterial. Log_2_ fold change < 0 represents the relative abundance of taxa decreased, Log_2_ fold change > 0 represents the relative abundance of taxa increased. CK represents treatment with blank control, BR represents treatment with broccoli residues. EJ-1 represents susceptible cultivar for CVW, J863 represents resistant cultivar for CVW. Asterisk represents significantly different by Duncan’s multiple range test at *P* < 0.05.

Among all sequences, unknown sequences were classified as “others”. The dominant bacterial phyla were Proteobacteria, Actinobacteria, Acidobacteria, Gemmatimonadetes, Chloroflexi, Bacteroidetes, Planctomycetes, Rokubacteria, Nitrospirae, Verrucomicrobia, Latescibacteria, Firmicutes and Patescibacteria, and these phyla accounted for more than 95% of the total sequences in each sample. The fold changes in the relative abundances of the dominant bacterial taxa associated with different cotton cultivars in BR treatment were compared at the phylum level (Figure 5A). The relative abundances of Actinobacteria, Gemmatimonadetes, Rokubacteria, Nitrospirae, Verrucomicrobia and Firmicutes for cultivar EJ-1, the susceptible cultivar, were increased in BR treatment. While for cultivar J863, the resistant cultivar, the relative abundances of Gemmatimonadetes, Chloroflexi, Planctomycetes, Rokubacteria, Verrucomicrobia, Latescibacteria and Firmicutes were increased (Figure 5A). Furthermore, analysis of variance showed the differences in the mean proportions of Proteobacteria, Bacteroidetes, Rokubacteria, Latescibacteria, Firmicutes and Patescibacteria (Figure 5B). The relative abundances of Gemmatimonadetes, Rokubacteria, Verrucomicrobia and Firmicutes were increased in susceptible and resistant cultivars, respectively. Firmicutes was the most fold changes (5.66) in susceptible cultivar EJ-1, while that for Verrucomicrobia (1.59) in resistant cultivar J863.

**FIGURE 6:**
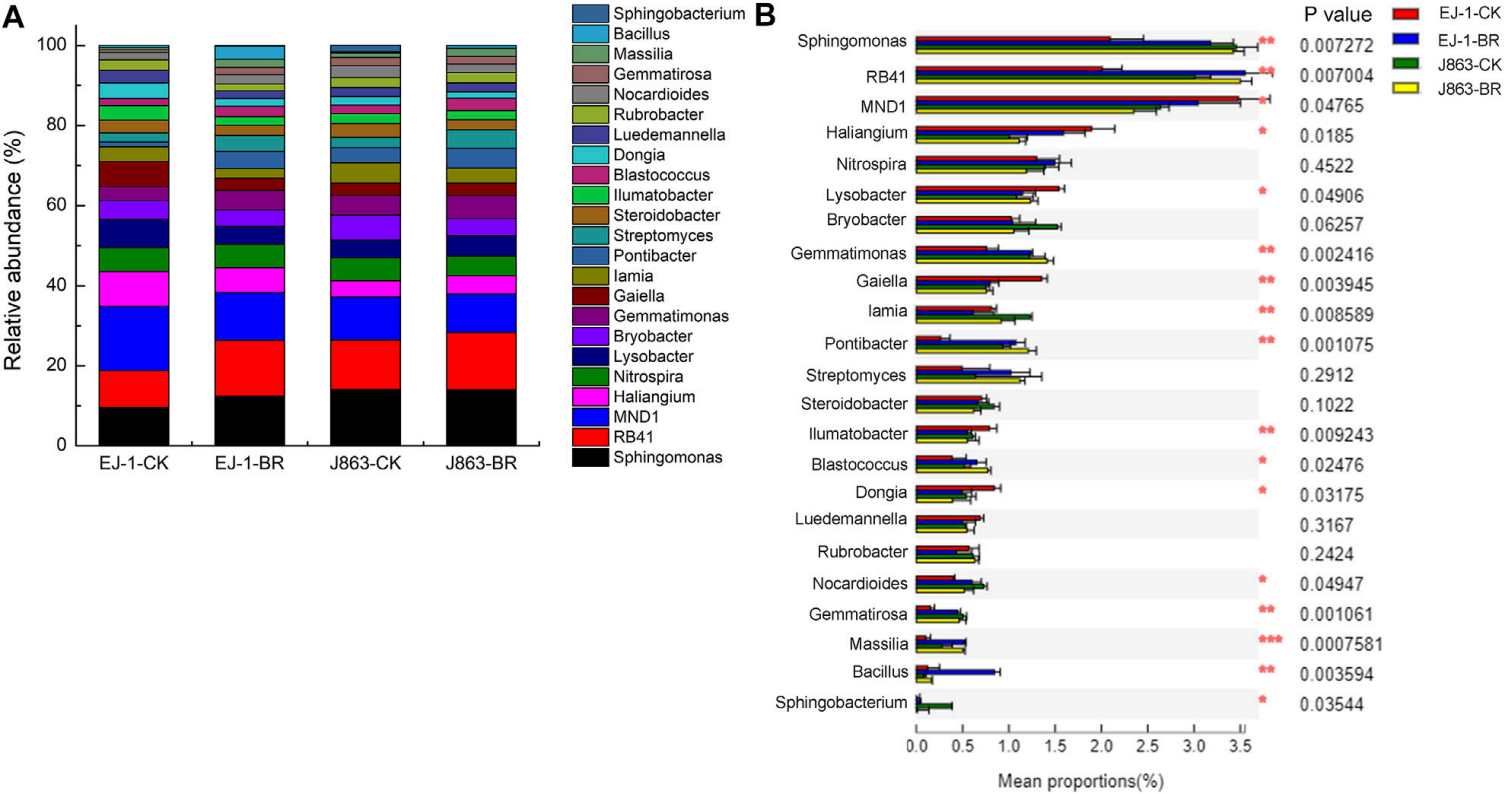
Changes in the relative abundances of the bacterial taxa at the genus level under different treatments. **(A)** Relative abundance of bacterial **(B)** Analysis of significant differences in mean proportion of bacterial. CK represents treatment with blank control, BR represents treatment with broccoli residues. EJ-1 represents susceptible cultivar for CVW, J863 represents resistant cultivar for CVW.

In addition, the top 23 dominant bacterial genera (cultured and at least one group in two groups average relative abundance>0.5%) were *Sphingomonas*, *RB41*, *MND1*, *Haliangium*, *Nitrospira*, *Lysobacter*, *Bryobacter*, *Gemmatimonas*, *Gaiella*, *Iamia*, *Pontibacter*, *Streptomyces*, *Steroidobacter*, *Ilumatobacter*, *Blastococcus*, *Dongia*, *Luedemannella*, *Rubrobacter*, *Nocardioides*, *Gemmatirosa*, *Massilia*, *Bacillus* and *Sphingobacterium* (Figure 6A). Furthermore, analysis of variance showed that the differences in the mean proportions of *Sphingomonas*, *RB41*, *MND1*, *Haliangium*, *Lysobacter*, *Gemmatimonas*, *Gaiella*, *Iamia*, *Pontibacter*, *Ilumatobacter*, *Blastococcus*, *Dongia*, *Nocardioides*, *Gemmatirosa*, *Massilia*, *Bacillus* and *Sphingobacterium* (Figure 6B). In terms of susceptible cultivar EJ-1, the relative abundances of *Sphingomonas*, *RB41*, *Gemmatimonas*, *Pontibacter*, *Streptomyces*, *Blastococcus*, *Nocardioides*, *Gemmatirosa*, *Massilia*, *Bacillus* and *Sphingobacterium* in BR treatment were increased by 29.46%, 50.95%, 39.23%, 49.67%, 75.91%, 44.95%, 28.41%, 151.74%, 338.52%, 486.83% and 710.03%, respectively. For resistant cultivar J863, the relative abundances of *RB41*, *Haliangium*, *Lysobacter*, *Gemmatimonas*, *Pontibacter*, *Streptomyces*, *Blastococcus*, *Massilia* and *Bacillus* in BR treatment were increased by 16.60%, 11.08%, 14.30%, 16.05%, 29.68%, 75.69%, 49.73%, 82.26% and 106.84%, respectively. Among them, the relative abundances of *RB41*, *Gemmatimonas*, *Pontibacter*, *Streptomyces*, *Blastococcus*, *Massilia* and *Bacillus* were increased by BR treatment in susceptible and resistant cultivars.

### Comparison of fungal community composition

**FIGURE 7:**
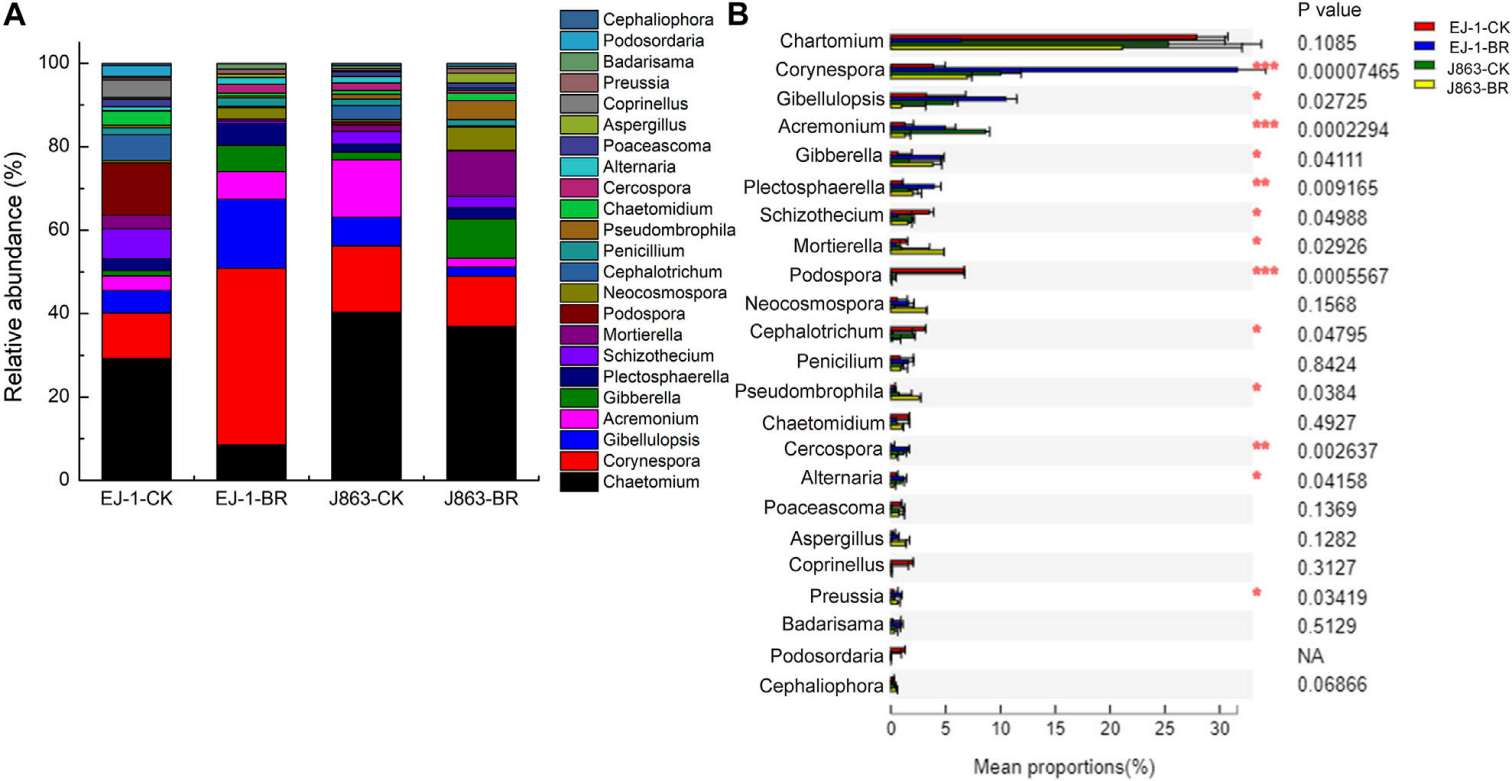
Changes in the relative abundances of the fungal taxa at the genus level in under different treatments. **(A)** Relative abundance of fungal **(B)** Analysis of significant differences in mean proportion of fungal. CK represents treatment with blank control, BR represents treatment with broccoli residues. EJ-1 represents susceptible cultivar for CVW, J863 represents resistant cultivar for CVW.

Ascomycota, Basidiomycota and Mortierellomycota were the dominant fungal phyla in different treatments. There was no significantly difference in susceptible and resistant cultivars in BR treatment (Supplementary Figure S1). At the genus level, the top 23 dominant fungal genera were *Chaetomium*, *Corynespora*, *Gibellulopsis*, *Acremonium*, *Gibberella*, *Plectosphaerella*, *Schizothecium*, *Mortierella*, *Podospora*, *Neocosmospora*, *Cephalotrichum*, *Penicillium*, *Pseudombrophila*, *Chaetomidium*, *Cercospora*, *Alternaria*, *Poaceascoma*, *Aspergillus*, *Coprinellus*, *Preussia*, *Badarisama*, *Podosordaria* and *Cephaliophora* (Figure 7A). Furthermore, analysis of variance showed that the differences in the mean proportions of *Corynespora*, *Gibellulopsis*, *Acremonium*, *Gibberella*, *Plectosphaerella*, *Schizothecium*, *Mortierella*, *Podospora*, *Cephalotrichum*, *Pseudombrophila*, *Cercospora*, *Alternaria* and *Preussia* (Figure 7B). In terms of susceptible cultivar EJ-1, the relative abundances of *Corynespora*, *Gibellulopsis*, *Acremonium*, *Gibberella*, *Plectosphaerella*, *Cercospora*, *Alternaria* and *Preussia* in BR treatment were increased, while others were decreased. For resistant cultivar J863, the relative abundances of *Gibberella*, *Plectosphaerella*, *Mortierella*, *Pseudombrophila* and *Preussia* were increased. Among them, the relative abundances of *Gibberella*, *Plectosphaerella*, *Alternaria* and *Preussia* were increased in BR treatment between susceptible and resistant cultivars, while the relative abundances of *Schizothecium*, *Podospora*, *Cephalotrichum* were decreased, respectively.

### Effect of BR on microbial community function in rhizosphere

**FIGURE 8:**
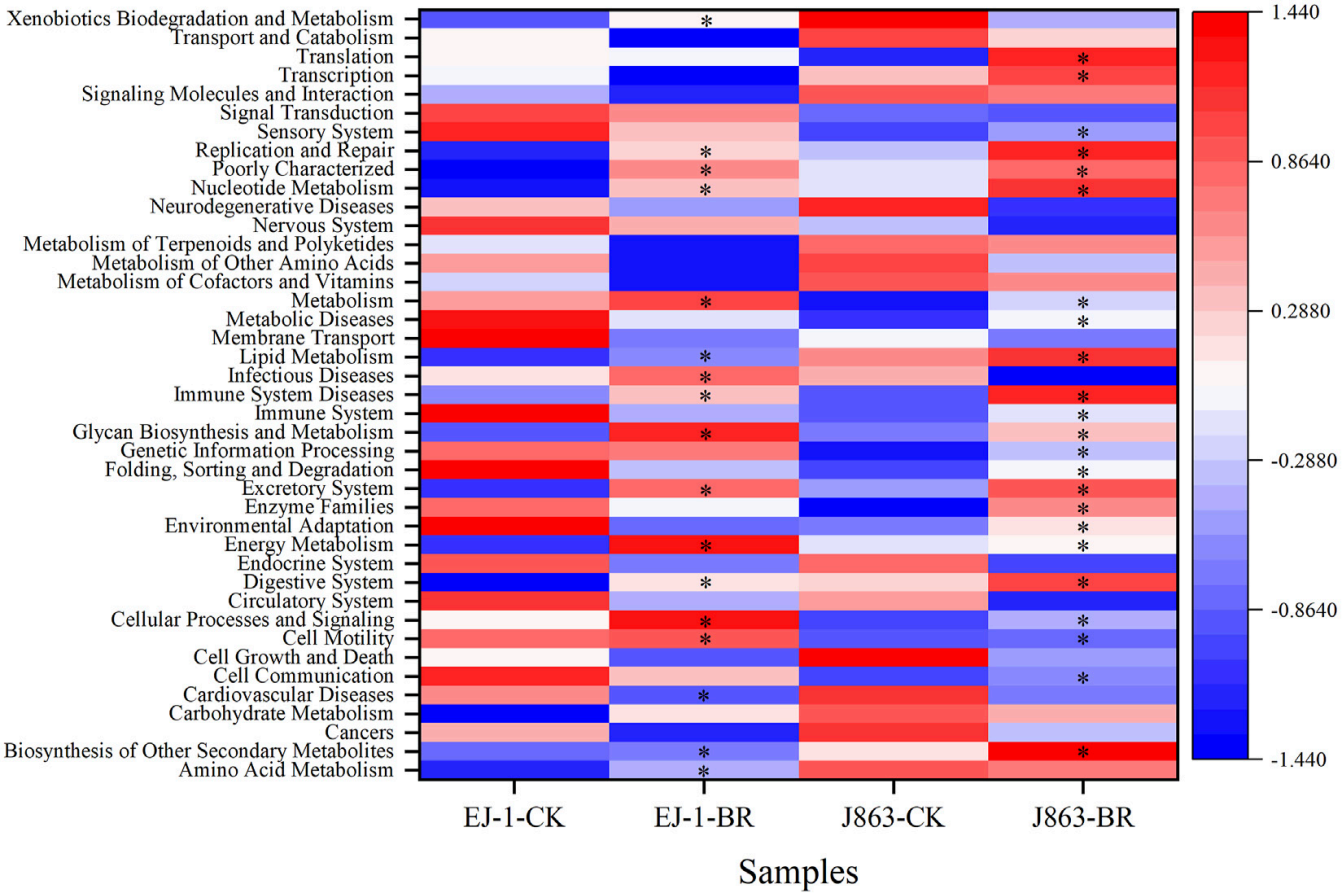
A heatmap of the predicted KEGG functional profiles (KEGG level 2) of bacterial microbiota across all samples. * represents functional pathways enrichment. CK represents treatment with blank control, BR represents treatment with broccoli residues. EJ-1 represents susceptible cultivar for CVW, J863 represents resistant cultivar for CVW.

To study the effect of BR on soil bacterial function, we used PICRUSt to perform bacterial function prediction analysis. Through comparisons with the KEGG database, 6 categories of biological metabolic pathways at level 1 were obtained, including metabolism, genetic information processing, environmental information processing, cellular processes, organ systems, and human diseases (Table 2). Among these pathways, metabolism, genetic information processing, and environmental information processing were the primary components, accounting for 51.50%–51.77%, 16.25%–16.32% and 12.85%–13.06%, respectively. The pathways of environmental information processing in different cultivars were downregulated in BR treatment compared with CK. The pathway of metabolism in susceptible cultivar EJ-1 was enriched in BR treatment, while the opposite tendency was obtained in resistant cultivar J863. In addition, the analysis of the functional pathways at level 2 of the predicted genes showed that it consisted of 41 subfunctions. For cultivar EJ-1, 17 pathways, including amino acid metabolism (e.g., tyrosine metabolism), carbohydrate metabolism (e.g., pentose and glucuronate interconversions, fructose, mannose, starch and sucrose metabolism), glycan biosynthesis and metabolism (e.g., N-glycan biosynthesis), lipid metabolism (e.g., sphingolipid, linoleic acid, steroid biosynthesis) were enriched in BR treatment compared with CK, while for cultivar J863, 23 pathways were enriched in BR treatment compared with CK (Figure 8).

**TABLE 2:** The proportion of predicted functional profiles in different treatments (Pathway level 1).

Pathway level1	EJ-1-CK (%)	EJ-1-BR (%)	J863-CK (%)	J863-BR (%)
Cellular processes	4.13	4.13	4.06	4.05
Environmental information processing	13.06	12.87	12.91	12.85
Genetic information processing	16.27	16.27	16.25	16.32
Human diseases	0.89	0.88	0.90	0.88
Metabolism	51.50	51.63	51.77	51.73
Organismal systems	0.81	0.80	0.80	0.80

**FIGURE 9:**
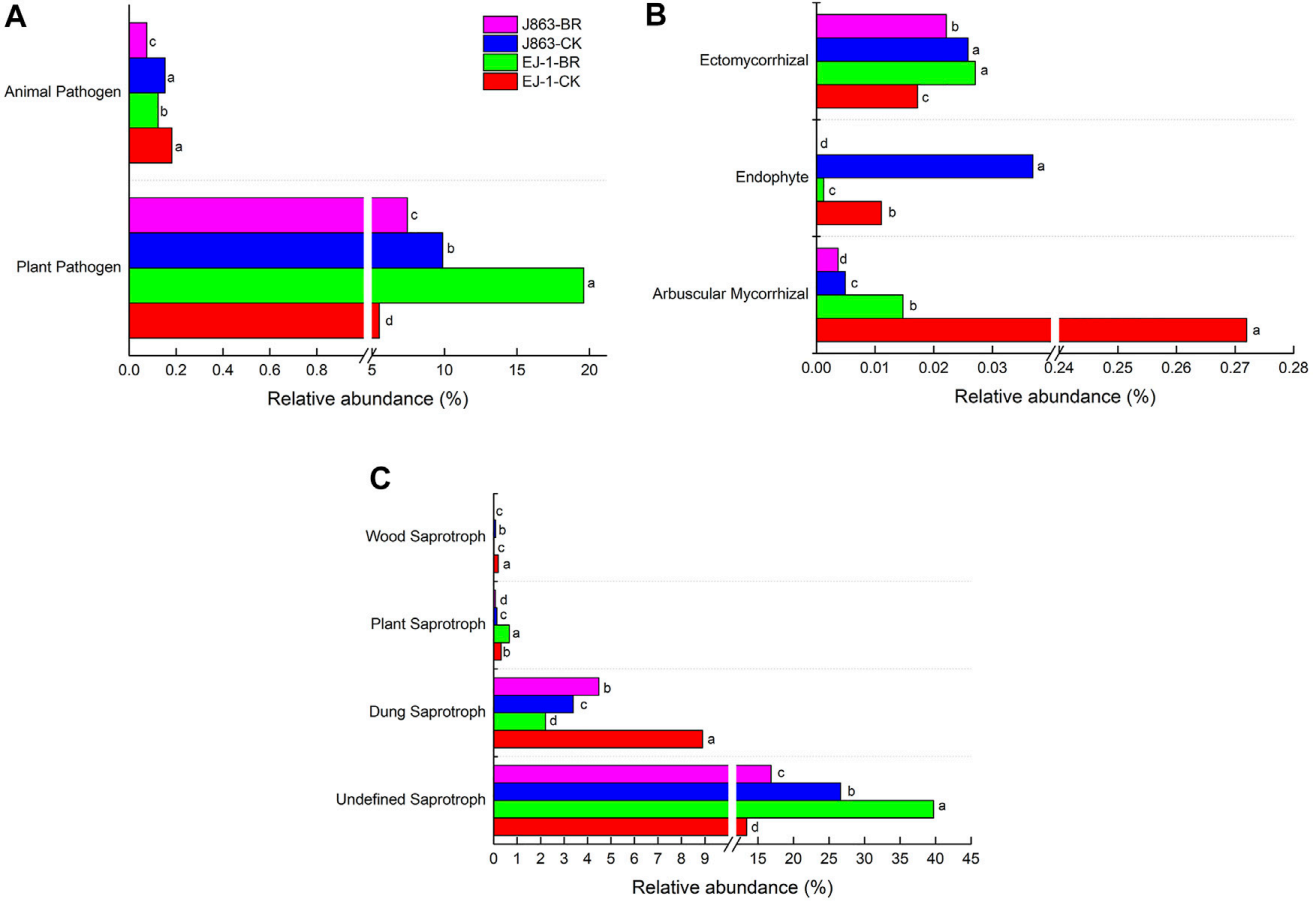
The relative abundance of mainly guilds assigned by FUNGuild for fungal communities. Different letters indicate significant difference between treatments detected by Student’s-T test (*P* < 0.05). **(A)** pathotroph trophic mode **(B)** symbiotroph trophic mode **(C)** saprotroph trophic mode.

FUNGuild was used to predict the nutritional and functional groups of the fungal communities with different treatments. The results showed that the fungi community was divided into nine trophic mode groups. The fungal community was screened to 484 identifiable species, which included saprotroph (43.80%), pathotroph-saprotroph-symbiotroph (16.32%), pathotroph (14.88%), symbiotroph (8.68%), pathotroph-saprotroph (6.20%), saprotroph-symbiotroph(6.20%), pathogen-saprotroph-symbiotroph (1.45%), pathotroph-symbiotroph (2.27%) and saprotroph-pathotroph-symbiotroph (0.21%) representing the general abundance of each nutrition method in the identified community (Supplementary Figure S2). Among them, pathotrophs, saprotrophs, and symbiotrophs were the major components. The pathotrophic mode groups primarily consisted of plant pathogens (not represent a specific specie) and animal pathogens. The relative abundances of plant pathogens in susceptible cultivar EJ-1 samples in BR treatment were higher than those observed in the control, while that in resistant cultivar J863 samples were significantly lower than that observed in the control. In addition, the relative abundances of animal pathogens in cultivars EJ-1 and J863 samples in BR treatment were also significantly lower than that observed in the control (Figure 9A). In terms of symbiotrophic mode groups, except ectomycorrhizal, the relative abundances of arbuscular mycorrhizal and endophyte in cultivars EJ-1 and J863 samples in BR treatment were significantly lower than that observed in the control (Figure 9B). For the mode groups of saprotroph, including undefined saprotroph, dung saprotroph, plant saprotroph and wood saprotroph. The relative abundance of dung saprotroph in cultivar EJ-1 samples in BR treatment was 2.21%, lower than that observed in the control (8.90%), while in cultivar J863 samples it was 4.48%, higher than that observed in the control (3.38%). However, the relative abundance of plant saprotroph in cultivar EJ-1 samples in BR treatment was 0.66%, higher than that observed in the control (0.31%), while in cultivar J863 samples it was 0.07%, lower than that observed in the control (0.14%) (Figure 9C).

## Discussion

### Evaluation of control effect of BR on CVW

The use of crop residues is an important method associated with the suppression of verticillium wilt, such as those of broccoli, buckwheat, canola, mustard, and sweet corn (P. Inderbitzin et al., 2018; W.S; Zhao et al., 2019; 2021; B.E; Wiggins and Kinkel, 2005; D.L; Wheeler and Johnson, 2016). Our previous study showed that incorporation of BR into soil could reduce the incidence of verticillium wilt of summer-sowing-cotton, and the control effect on CVW reached 70.77% under the broccoli and cotton double cropping cultivation mode (W.S. Zhao et al., 2019). In this study, cotton cultivars with different verticillium wilt resistance levels were chosen to evaluate the effect of BR on the occurrence of verticillium wilt of spring-sowing-cotton. The results showed that the control effect of BR on susceptible and resistant cultivars were 58.49% and 85.96%, respectively. Meanwhile, it could significantly reduce the populations of *V. dahliae* in soil. Our results are consistent with those from previous studies on disease in cauliflower, eggplant, and potato in BR treatment (W.S. Zhao et al., 2019; K.V; Subbarao et al., 1999; K; Ikeda et al., 2015; J.R; Davis et al., 2010). Therefore, the application of BR provides a new method and idea for sustainable prevention and control of CVW in different planting patterns.

### Effect of BR on soil microbial community structure in rhizosphere of different cultivars resistant to CVW

Development of an effective strategy for application of plant residues is critically dependent on our understanding of several soil microbiological and their interactions (X.F. Yuan et al., 2021; C.F; Zhang et al., 2021). There were different opinions on the relationships between microbial community diversity and disease incidence (L.R. Bulluck et al., 2002; Z.Z; Shen et al., 2014). Many studies showed that the diversity and composition of soil microbial community were related to the occurrence of soil-borne diseases (W.S. Zhao et al., 2021; L.R; Bulluck et al., 2002; A; Gamliel et al., 2000). Previous studies suggested that greater bacterial diversity in soils promoted resistance to plant disease (L. Shi et al., 2017). However, some scholars reported that there were no significant correlations between bacterial community diversity and banana fusarium wilt following the application of bioorganic fertilizer (Z.Z. Shen et al., 2014). In the present study, the bacterial diversity indices significantly increased after the application of BR, however, the fungal diversity indices significantly decreased. Meanwhile, the results of β-diversity showed that the bacterial community structures in rhizosphere among cultivars were significantly different, and the bacterial community structures were changed and located in the same quadrant in BR treatment, indicating that the bacterial community structures tended to be consistent and developed toward the direction of healthy soil in BR treatment. This result was consistent with the previous studies on the changes in bacterial community structure in the rhizospheric soil of eggplant (P. Inderbitzin et al., 2018). In terms of fungi community structure, there were no significant differences in the rhizosphere among cultivars themselves, however, it was changed in BR treatment compared with CK. Therefore, it was suggested that BR treatment played an important role in regulated microbial community structures in cotton rhizosphere. In addition, the fact that of the composition of the rhizosphere microbiota were influenced by cultivars, soil types, health status and growing stages of plant (W.S. Zhao et al., 2020; X.M; Xu et al., 2010; Z.Q; Huang et al., 2021).

### Effects of BR on key microbiota of soil microbial community in cotton rhizosphere

Proteobacteria, Actinobacteria, Bacteroidetes, Firmicutes, and Acidobacteria were the five dominant phyla in rhizosphere of eggplant (P. Inderbitzin et al., 2018). Actinobacteria was considered a plant growth promoting bacteria and could produce diverse bioactive compounds (T. Elsayed, et al., 2020). Some bacteria that belong to Proteobacteria, such as *Sphingomonas* sp., could protect tomato against gray mold (J. Enya et al., 2007) and rice seedling disease (T.B. Adhikari et al., 2001), reduce nitrate and enhance total nitrogen removal in biological denitrification processes (W. Xing et al., 2019). Acidobacteria might be potential biological control agents for bacterial wilt and could promote plant growth (C.T. Yin et al., 2013; Y.H; Xiao et al., 2018). Some members in the phylum Gemmatimonadetes might be a potential indicator of soil health or halophilic (M.S. Hahm et al., 2016). Acidobacteria and Gemmatimonadetes could also participate in carbon, nitrogen, and sulfur biogeochemical cycles of the rhizosphere soil (S. Kalam et al., 2020; C.L.M; Khodadad et al., 2011). Firmicutes played an important role in the biological control of plant disease and plant growth promoting (Y.X. Feng et al., 2019; M.C; Azabou et al., 2020; S.Z; Li et al., 2005). Compared with the blank control, the recruitment abilities of Actinobacteria, Gemmatimonadetes, Firmicutes and Rokubacteria in susceptible cultivar EJ-1 were increased after application of BR treatment, while the recruitment abilities of Gemmatimonadetes, Firmicutes and Verrucomicrobia in resistant cultivar J863 were increased. At the genus level, *RB41* could contribute to N assimilation (M.A. Meier et al., 2021). *Gemmatimonas* was related to the metabolism and transformation of nitrogen (Z. Liu et al., 2021). *Blastococcus* was associated with the assimilation of carbon and involved in the metabolizing the intermediate products of residue decomposition (F.L. Fan et al., 2014). *Streptomyces* and *Bacillus* were used as biological control agents against plant pathogens (P. Inderbitzin et al., 2018; Z.Q; Huang et al., 2021). In our study, the relative abundances of *Sphingomonas*, *Nocardioides*, *Gemmatirosa* and *Sphingobacterium* for susceptible cultivar EJ-1 were increased in BR treatment, while that for *Haliangium*, *Lysobacter*, *Gaiella*, *Luedemannella* and *Rubrobacter* were decreased, respectively. However, these relative abundances of above microbiota in resistant cultivar J863 showed opposite results in BR treatment compared with that of susceptible cultivar EJ-1 (Supplementary Table S1). In addition, the relative abundances of *RB41*, *Gemmatimonas*, *Pontibacter*, *Streptomyces*, *Blastococcus*, *Massilia* and *Bacillus* were increased for different cultivars in BR treatment, while that for *MND1*, *Nitrospira*, *Bryobacter*, *Iamia*, *Steroidobacter*, *Ilumatobacter* and *Dongia* were decreased, respectively (Supplementary Table S1).

In terms of fungi, Ascomycota, Basidiomycota and Mortierellomycota were the dominant phyla, and the relative abundances of soil fungal communities at phyla level were not significantly affected by BR treatment. At the genus level, *Penicillium* showed good inhibitory effect on peach blight and growth promotion (G.N. Belofsky et al., 1998; A.D; Cal et al., 1990; M.M; Hossain et al., 2007). In our study, the relative abundances of *Corynespora*, *Gibellulopsis*, *Acremonium*, *Penicillium*, *Cercospora*, *Alternaria* and *Badarisama* in susceptible cultivar EJ-1 were increased in BR treatment, while that for *Chaetomium*, *Mortierella*, *Pseudombrophila*, *Chaetomidium*, *Poaceascoma* and *Cephaliophora* were decreased, respectively. Compared with susceptible cultivar EJ-1, these relative abundances of above microbiota in resistant cultivar J863 showed opposite results in BR treatment (Supplementary Table S2). In addition, the relative abundances of *Gibberella*, *Plectosphaerella*, *Neocosmospora*, *Aspergillus*, and *Preussia* were increased for different cultivars in BR treatment, while that for *Chaetomium*, *Schizothecium*, *Podospora*, *Cephalotrichum*, *Coprinellus* and *Podosordaria* were decreased, respectively (Supplementary Table S2). These results were roughly in line with the basic fact that BR treatment promoted the proportion of beneficial soil microbes and inhibited the proportion of pathogens to increase plant defense. Similar results showed in recent studies that microbial biocontrol agents treatment promoted beneficial microorganisms in soil and suppressed the pathogens (Z.Q. Huang et al., 2021).

### Variations in microbial functional metabolic genes after application of BR

PICRUSt analysis has been used to study bacterial functions and can predict the presence or absence of functional genes and their abundances (L. Jiang et al., 2016; J; Luo et al., 2017). Some studies indicated that energy metabolism could enhance resistance to banana fusarium wilt (N.V.D. Berg et al., 2007). The function of biosynthesis of other secondary metabolites could antagonistic to several soil-borne pathogens (L. Han et al., 2019). In our study, there was a significantly increase of energy metabolism and biosynthesis of other secondary metabolites after application of BR. PICRUSt was also used to predict the relative abundances of key genes in the C, N and *p* cycles (H. Ribeiro et al., 2018; Y; Chen et al., 2020). However, due to the limitations of using a 16 S rRNA dataset and taxonomy assignment method, the taxa annotation and its function were insufficient, therefore, further validation should be performed using metagenomics to better understand the function of the rhizosphere bacterial community after application of BR in the future studies.

Variations in the composition of fungal functional groups inferred by FUNGuild showed that BR treatment could influence nutrition mode (Figure 9). For instance, the trophic mode of pathotroph consisted of plant pathogens and animal pathogens, the relative abundance of plant pathogens for susceptible cultivar EJ-1 in BR treatment was higher than in CK treatment, while the opposite tendency was observed for resistant cultivar J863. In the present study, the populations of *V. dahliae* were decreased in BR treatment by qPCR analysis. Analysis of the composition of fungal communities showed that BR treatment could simultaneously increase proportions of *Gibellulopsis*, *Plectosphaerella*, *Gibberella* (Figure7B). *Plectosphaerella* sp. is well known as a pathogen of several plant species causing fruit, root and collar rot, and collapse (A. Carlucci et al., 2012). The genus of *Gibberella* could cause different plant diseases in tropical and subtropical areas of the world (C.S. Lima et al., 2009; W.G.D Fernando et al., 1997). For instance, mango malformation disease was mainly caused by the pathogen of *Gibberella fujikuroi* species complex in Brazilian (C.S. Lima et al., 2009). As trophic mode of symbiotroph, arbuscular mycorrhizal fungi (AMF) could form a symbiotic relationship with more than 80% of terrestrial plants, enhancing the absorption capacity of root systems and providing nutrients for plant growth (A. Berruti et al., 2016). However, in our study, the relative abundances of AMF and endophyte fungi were significantly lower than that in CK treatment, suggesting that symbiotroph was not dominant trophic mode in BR treatment. In terms of saprotroph trophic modes, some studies showed that saprophytic fungi played important roles in decomposing organic matter and nutrient cycling and were the primary decomposers of dead or aged plants in soil (Y. Chen et al., 2020; L.A; Phillips et al., 2014). In this study, the relative abundances of undefined saprotroph (39.72%) and plant saprotroph (0.66%) in cultivar EJ-1 in BR treatment were higher than that of CK treatment (13.44%, 0.31%), while the opposite tendencies were observed in cultivar J863. Analysis of the composition of fungal communities showed that BR treatment could simultaneously enrich of *Schizothecium*, *Preussia* in different cultivars (Figure7B). Due to FUNGuild analysis was based on preexisting literature and data, there were some limitations for analyze the functions of fungi to some extent. Thus, additional in-depth studies on soil fungal functional groups are also needed to further investigate the function of the rhizosphere fungal community in BR treatment.

Root exudates could act as signal molecules that regulated the community structure and function of the rhizosphere microbiome (J. Panichikkal and Krishnankutty, 2021). Therefore, the possible reason was that in addition to the differences of the root exudates of the cultivars themselves, BR changed the proportion of root exudates, which could cause different microbial communities to be recruited. Therefore, the microbial community was changed then to enter a new balance in BR treatment, and this change was the result of the interactions between BR treatment and indigenous microbes (Z.Q. Huang et al., 2021; J; Sylla et al., 2013). Moreover, the interactions among BR treatment with indigenous rhizosphere microbes and plant were complicated, which due to it might increase beneficial or inhibit harmful microbes. In our study, BR treatment could suppress plant disease and promote plant health, which should not simply be attributed to a single bacterial taxon, but was most likely regulated by microbial consortia, which was similar result observed in recent studies (L. Shi et al., 2017; M.A; Cucu et al., 2020). Some researchers reported that direct applications of potentially beneficial microbes often resulted in poor disease suppression due to their low survival and colonization in soil (T. Saravanan et al., 2003; B; Lugtenberg and Kamilova, 2009). Therefore, the future research will focus on the acquisition of beneficial microorganisms, the construction of synthetic communities and evaluation the effects of these beneficial microorganisms on the incidence of CVW.

In conclusion, the incidence of CVW and the populations of *V. dahliae* in the rhizosphere of different cotton resistant cultivars were decreased in BR treatment. Meanwhile, partial plant biomass and cotton yields were increased. The bacterial diversities were significantly increased in BR treatment, but the opposite tendency for fungal diversities in different resistant cultivars. There were significantly differences in the ability to recruit beneficial microorganisms in BR treatment between susceptible and resistant cultivars. The relative abundances of key microbes were changed, which effected on CVW in BR treatment. These results provide important information necessary for a better understanding of microbial community structure and function in rhizosphere soil in BR treatment. In the future, potential beneficial synthetic communities will be required to further explore to control CVW.

## Data Availability

Publicly available datasets were analyzed in this study. The raw sequences data were deposited at the National Center for Biotechnology Information (NCBI) under accession numbers PRJNA734729 and PRJNA894483.
