# Supplementary material for: Effect of incorporation of broccoli residues into soil on occurrence of verticillium wilt of spring-sowing-cotton and on rhizosphere microbial communities structure and function
Source: Front Bioeng Biotechnol. 2023 Jan 24;11:1115656. doi: 10.3389/fbioe.2023.1115656 (PMC9902944; doi:10.3389/fbioe.2023.1115656)

Table S1 Relative abundance and change rate of bacterial community at genus level in BR treatment

| Genus | Relative abundance (%) | | Change rate(%) | Relative abundance (%) | | Change rate(%) |
| --- | --- | --- | --- | --- | --- | --- |
|  | EJ-1-CK | EJ-1-BR |  | J863-CK | J863-BR |  |
| Sphingomonas | 9.62 | 12.45 | 29.46 | 14.11 | 14.01 | -0.69 |
| RB41 | 9.22 | 13.92 | 50.95 | 12.29 | 14.33 | 16.60 |
| MND1 | 15.99 | 11.91 | -25.49 | 10.77 | 9.61 | -10.75 |
| Haliangium | 8.69 | 6.23 | -28.37 | 4.10 | 4.55 | 11.08 |
| Nitrospira | 5.97 | 5.85 | -1.97 | 5.68 | 4.87 | -14.24 |
| Lysobacter | 7.07 | 4.49 | -36.42 | 4.42 | 5.05 | 14.30 |
| Bryobacter | 4.71 | 4.07 | -13.54 | 6.22 | 4.31 | -30.67 |
| Gemmatimonas | 3.49 | 4.85 | 39.23 | 4.99 | 5.79 | 16.05 |
| Gaiella | 6.22 | 3.10 | -50.11 | 3.07 | 3.10 | 0.93 |
| Iamia | 3.72 | 2.41 | -35.23 | 5.01 | 3.74 | -25.23 |
| Pontibacter | 1.20 | 4.20 | 249.67 | 3.81 | 4.94 | 29.68 |
| Streptomyces | 2.27 | 4.00 | 75.91 | 2.61 | 4.58 | 75.69 |
| Steroidobacter | 3.23 | 2.60 | -19.39 | 3.42 | 2.53 | -26.15 |
| Ilumatobacter | 3.63 | 2.16 | -40.39 | 2.49 | 2.26 | -9.19 |
| Blastococcus | 1.76 | 2.56 | 44.95 | 2.11 | 3.15 | 49.73 |
| Dongia | 3.86 | 1.94 | -49.81 | 2.20 | 1.60 | -27.35 |
| Luedemannella | 3.17 | 1.96 | -38.20 | 2.18 | 2.23 | 2.55 |
| Rubrobacter | 2.61 | 1.69 | -35.24 | 2.51 | 2.58 | 3.03 |
| Nocardioides | 1.83 | 2.35 | 28.41 | 2.97 | 2.11 | -28.84 |
| Gemmatirosa | 0.69 | 1.75 | 151.74 | 2.07 | 1.90 | -8.27 |
| Massilia | 0.46 | 2.03 | 338.51 | 1.13 | 2.05 | 82.26 |
| Bacillus | 0.56 | 3.30 | 486.83 | 0.31 | 0.65 | 106.84 |
| Sphingobacterium | 0.02 | 0.18 | 710.03 | 1.56 | 0.04 | -97.48 |

Table S2 Relative abundance and change rate of fungal community at genus level in BR treatment

| Genus | Relative abundance (%) | | Change rate(%) | Relative abundance (%) | | Change rate(%) |
| --- | --- | --- | --- | --- | --- | --- |
|  | EJ-1-CK | EJ-1-BR |  | J863-CK | J863-BR |  |
| Chaetomium | 29.21 | 8.55 | -70.71 | 40.29 | 36.88 | -8.47 |
| Corynespora | 10.94 | 42.36 | 287.25 | 15.93 | 12.11 | -23.96 |
| Gibellulopsis | 5.41 | 16.54 | 205.61 | 6.93 | 2.13 | -69.20 |
| Acremonium | 3.51 | 6.64 | 89.12 | 13.73 | 2.16 | -84.30 |
| Gibberella | 1.29 | 6.26 | 386.12 | 1.86 | 9.44 | 406.29 |
| Plectosphaerella | 2.85 | 5.26 | 84.48 | 1.87 | 2.67 | 42.90 |
| Schizothecium | 7.18 | 0.55 | -92.38 | 3.12 | 2.69 | -13.91 |
| Mortierella | 3.23 | 0.47 | -85.53 | 1.52 | 10.96 | 619.52 |
| Podospora | 12.55 | 0.03 | -99.76 | 0.72 | 0.09 | -88.07 |
| Neocosmospora | 0.53 | 2.71 | 415.78 | 0.54 | 5.61 | 935.02 |
| Cephalotrichum | 6.22 | 0.27 | -95.70 | 3.36 | 0.23 | -93.16 |
| Penicillium | 1.65 | 2.14 | 29.25 | 1.56 | 1.56 | -0.02 |
| Pseudombrophila | 0.63 | 0.44 | -30.07 | 1.18 | 4.53 | 282.71 |
| Chaetomidium | 3.34 | 0.65 | -80.53 | 0.85 | 1.78 | 109.43 |
| Cercospora | 0.16 | 2.16 | 1259.36 | 1.86 | 0.77 | -58.84 |
| Alternaria | 0.89 | 1.55 | 74.35 | 1.49 | 0.44 | -70.33 |
| Poaceascoma | 1.87 | 0.11 | -94.26 | 1.21 | 1.25 | 3.01 |
| Aspergillus | 0.39 | 0.76 | 96.85 | 0.16 | 2.33 | 1333.52 |
| Coprinellus | 4.14 | 0.00 | -99.88 | 0.13 | 0.00 | -96.57 |
| Preussia | 0.49 | 1.20 | 146.63 | 0.46 | 1.11 | 140.45 |
| Badarisama | 0.37 | 1.23 | 229.14 | 0.65 | 0.51 | -22.44 |
| Podosordaria | 2.64 | 0.01 | -99.63 | 0.02 | 0.00 | -100.00 |
| Cephaliophora | 0.52 | 0.12 | -76.80 | 0.53 | 0.75 | 39.51 |

**Fig. S1** Analysis of significant differences in mean proportion of dominant fungal. CK represents treatment with blank control, BR represents treatment with broccoli residues. EJ-1 represents susceptible cultivar for CVW, J863 represents resistant cultivar for CVW. Asterisk represents significantly different by Duncan’s multiple range test at P < 0.05.


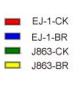

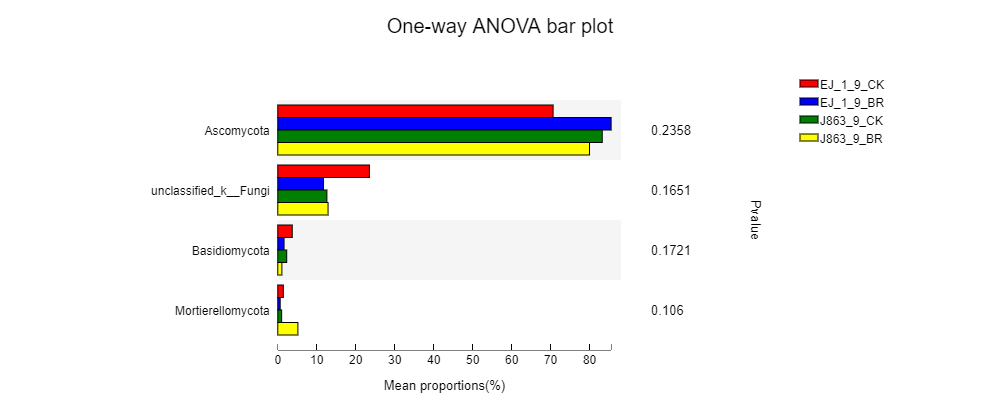


**Fig. S2** The relative abundance of trophic modes assigned by FUNGuild for fungal communities.


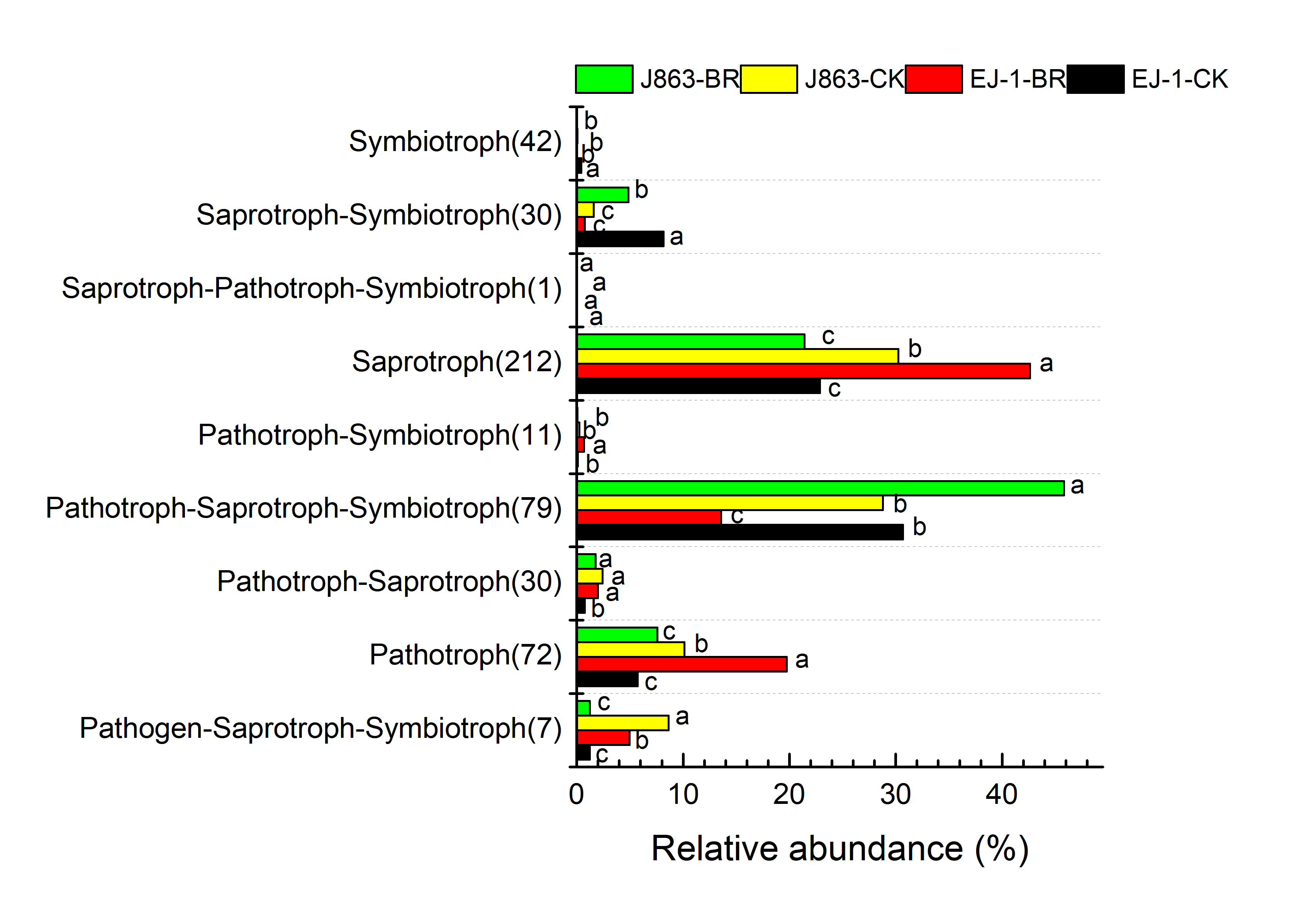

Supplement: Supplementary file 1 [file DataSheet1.doc]
