# Supplementary figures and images for: Effect of incorporation of broccoli residues into soil on occurrence of verticillium wilt of spring-sowing-cotton and on rhizosphere microbial communities structure and function
Source: Front Bioeng Biotechnol. 2023 Jan 24;11:1115656. doi: 10.3389/fbioe.2023.1115656 (PMC9902944; doi:10.3389/fbioe.2023.1115656)

**Fig. S2** The relative abundance of trophic modes assigned by FUNGuild for fungal communities.

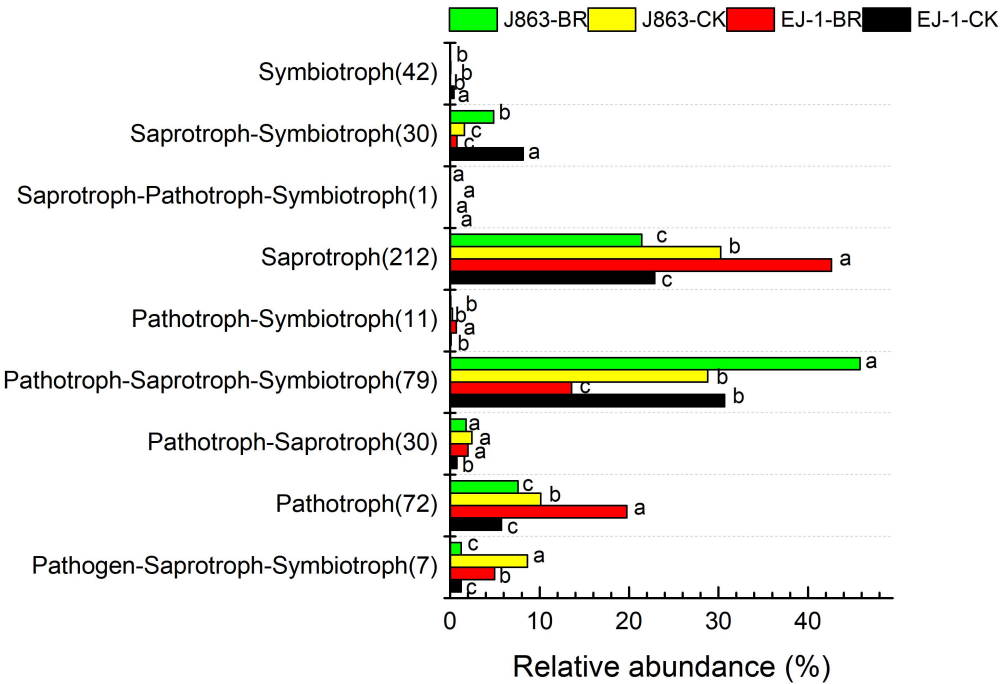

Supplement: Supplementary file 2 [file Image2.pdf]
