# Supplementary material for: Effect of incorporation of broccoli residues into soil on occurrence of verticillium wilt of spring-sowing-cotton and on rhizosphere microbial communities structure and function
Source: Front Bioeng Biotechnol. 2023 Jan 24;11:1115656. doi: 10.3389/fbioe.2023.1115656 (PMC9902944; doi:10.3389/fbioe.2023.1115656)
Supplement: Supplementary file 3 [file Table1.doc]

Table S1 Relative abundance and change rate of bacterial community at genus level in BR treatment

| Genus | Relative abundance (%) | | Change rate(%) | Relative abundance (%) | | Change rate(%) |
| --- | --- | --- | --- | --- | --- | --- |
|  | EJ-1-CK | EJ-1-BR |  | J863-CK | J863-BR |  |
| Sphingomonas | 9.62 | 12.45 | 29.46 | 14.11 | 14.01 | -0.69 |
| RB41 | 9.22 | 13.92 | 50.95 | 12.29 | 14.33 | 16.60 |
| MND1 | 15.99 | 11.91 | -25.49 | 10.77 | 9.61 | -10.75 |
| Haliangium | 8.69 | 6.23 | -28.37 | 4.10 | 4.55 | 11.08 |
| Nitrospira | 5.97 | 5.85 | -1.97 | 5.68 | 4.87 | -14.24 |
| Lysobacter | 7.07 | 4.49 | -36.42 | 4.42 | 5.05 | 14.30 |
| Bryobacter | 4.71 | 4.07 | -13.54 | 6.22 | 4.31 | -30.67 |
| Gemmatimonas | 3.49 | 4.85 | 39.23 | 4.99 | 5.79 | 16.05 |
| Gaiella | 6.22 | 3.10 | -50.11 | 3.07 | 3.10 | 0.93 |
| Iamia | 3.72 | 2.41 | -35.23 | 5.01 | 3.74 | -25.23 |
| Pontibacter | 1.20 | 4.20 | 249.67 | 3.81 | 4.94 | 29.68 |
| Streptomyces | 2.27 | 4.00 | 75.91 | 2.61 | 4.58 | 75.69 |
| Steroidobacter | 3.23 | 2.60 | -19.39 | 3.42 | 2.53 | -26.15 |
| Ilumatobacter | 3.63 | 2.16 | -40.39 | 2.49 | 2.26 | -9.19 |
| Blastococcus | 1.76 | 2.56 | 44.95 | 2.11 | 3.15 | 49.73 |
| Dongia | 3.86 | 1.94 | -49.81 | 2.20 | 1.60 | -27.35 |
| Luedemannella | 3.17 | 1.96 | -38.20 | 2.18 | 2.23 | 2.55 |
| Rubrobacter | 2.61 | 1.69 | -35.24 | 2.51 | 2.58 | 3.03 |
| Nocardioides | 1.83 | 2.35 | 28.41 | 2.97 | 2.11 | -28.84 |
| Gemmatirosa | 0.69 | 1.75 | 151.74 | 2.07 | 1.90 | -8.27 |
| Massilia | 0.46 | 2.03 | 338.51 | 1.13 | 2.05 | 82.26 |
| Bacillus | 0.56 | 3.30 | 486.83 | 0.31 | 0.65 | 106.84 |
| Sphingobacterium | 0.02 | 0.18 | 710.03 | 1.56 | 0.04 | -97.48 |
