# Supplementary material for: Effect of incorporation of broccoli residues into soil on occurrence of verticillium wilt of spring-sowing-cotton and on rhizosphere microbial communities structure and function
Source: Front Bioeng Biotechnol. 2023 Jan 24;11:1115656. doi: 10.3389/fbioe.2023.1115656 (PMC9902944; doi:10.3389/fbioe.2023.1115656)

Fig. S1 Analysis of significant differences in mean proportion of dominant fungal. CK represents treatment with blank control, BR represents treatment with broccoli residues. EJ-1 represents susceptible cultivar for CVW, J863 represents resistant cultivar for CVW. Asterisk represents significantly different by Duncan's multiple range test at P < 0.05.

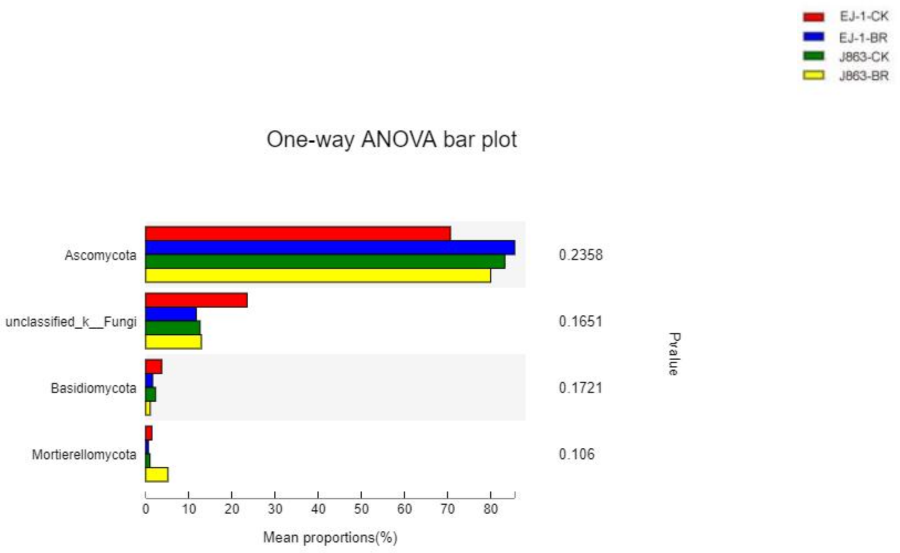

Supplement: Supplementary file 4 [file Image1.pdf]
